# NEIGHBORHOOD LIFE FOR PERSONS WITH DEMENTIA: A SYSTEMATIC REVIEW AND META-SYNTHESIS OF WALKING INTERVIEWS

**DOI:** 10.1093/geroni/igae098.2359

**Published:** 2024-12-31

**Authors:** Wenjin Wang, Xi Chen, Chanam Lee, Ziying Zhang

**Affiliations:** Texas A&M University, College Station, Texas, United States; Texas A&M University, College Station, Texas, United States; Texas A&M University, College Station, Texas, United States; Simon Fraser University, Burnaby, British Columbia, Canada

## Abstract

The number of persons with dementia (PwDs) is rising significantly, with most still residing in the community and spending much time within their neighborhoods. This study aims to provide a critical and comprehensive review of existing qualitative studies documenting the impact of neighborhood environments on PwDs’ attitudes and perceptions toward their neighborhood life. A systematic search of Web of Science, Pubmed, CINAHL Ultimate, APA PsycInfo, Academic Search Ultimate, and EMBASE was conducted. Empirical studies on community-dwelling PwDs that use walking interviews in the neighborhoods were included in the review/synthesis. Meta-synthesis guided data collection, quality assessment, and analysis. The search resulted in 17317 articles of which 17 met the eligibility criteria. They had a combined sample of 186 people with memory problems, mild cognitive impairment, or dementia. Preliminary results suggest three themes— (1) Physical environment: Land use, urban design, transportation, wayfinding and legibility, and outdoor interaction and comfort are integral for PwDs connecting with the neighborhood; (2) Social Environment: Care, support, and connections foster PwDs‘social health, while neighborhood engagement and belonging enhance their identity and quality of life; (3) Technological Environment: Access to and use of technology can bridge gaps in PwDs’ everyday communication and activity, facilitating their presence and engagement. This is the first effort to synthesize qualitative evidence obtained directly from community-dwelling PwDs who face additional challenges in maintaining a positive neighborhood life. The significant roles of the many environmental elements/features across the physical, social, and technology domains can guide future research and practice toward creating dementia-friendly communities.

